# Off-Axis Integral Cavity Carbon Dioxide Gas Sensor Based on Machine-Learning-Based Optimization

**DOI:** 10.3390/s24165226

**Published:** 2024-08-13

**Authors:** Pengbo Li, Guanyu Lin, Jianbo Chen, Jianing Wang

**Affiliations:** 1 Changchun Institute of Optics, Fine Mechanics and Physics, Chinese Academy of Sciences, Changchun 130033, China; lipengbo20@mails.ucas.ac.cn (P.L.);; 2 University of Chinese Academy of Sciences, Beijing 101408, China; 3 School of optoelectronic engineering, Changchun University of Science and Technology, Changchun 130012, China

**Keywords:** greenhouse gas, trace gas detection, off-axis integrating cavity output spectrum, machine learning

## Abstract

Accurately detecting atmospheric carbon dioxide is a vital part of responding to the global greenhouse effect. Conventional off-axis integral cavity detection systems are computationally intensive and susceptible to environmental factors. This study deploys an Extreme Learning Machine model incorporating a cascaded integrator comb (CIC) filter into the off-axis integrating cavity. It is shown that appropriate parameters can effectively improve the performance of the instrument in terms of lower detection limit, accuracy, and root mean square deviation. The proposed method is incorporated successfully into a monitoring station situated near an industrial area for detecting atmospheric carbon dioxide (CO_2_) concentration daily.

## 1. Introduction

Since the 1850s, the annual global mean temperature of the atmosphere has increased by over 1.4 °C. Additionally, the global mean sea level reached a record high as per the satellite record in 2023. The areas covered by ice and snow, known as the cryosphere, are experiencing historically low levels due to climate change. Many parts of the world have been affected by extreme heat as well [1,2]. Research indicates that human activities, particularly the emission of greenhouse gasses (GHS), are unequivocally responsible for global warming [1]. Greenhouse gasses, including water vapor, carbon dioxide (CO_2_), methane (CH_4_), nitrous oxide(N_2_O), and others, absorb the radiation wavelengths emitted by a planet, leading to the greenhouse effect. When considering concentration and global warming potential (GWP), the greenhouse effect of carbon dioxide ranks first among all greenhouse gasses. Its effective radiative forcing (ERF, in W∙m^−2^) in 2019 compared to 1850 was 2.012 [3]. Many studies have attempted to measure gas concentrations using spectroscopy methods, aiming to achieve advantages over traditional approaches such as electrochemical and chemical methods [4,5].

Various spectroscopy methods, such as Cavity Ring-Down Spectroscopy (CRDS) [6,7,8], Non-dispersive Infrared (NDIR) techniques [9,10], Optical Feedback Cavity Enhanced Absorption Spectroscopy (OF-CEAS) [11,12], and other spectroscopy techniques [12,13], are currently being utilized for detecting gas concentration. However, these methods suffer from poor environmental adaptability, complexity of the detection systems, and inadequate accuracy of detection, which act as key impediments to the large-scale utilization of these techniques. 

Off-Axis Integrated Cavity Output Spectroscopy (OA-ICOS) has been developed as a gas detection technology in 2001 [14]. This technique is commonly used by researchers to detect greenhouse gasses, pollutant gasses, dissolved gasses in water, and isotopes. Although the same absorption spectroscopy technique is used, unlike coaxial technology such as CRDS or CEAS, the optical system of OA-ICOS deliberately destroys the resonance state of the cell. The coaxiality of coaxial systems is susceptible to environmental factors and is highly demanding on the application environment. On the contrary, this approach, which is not based on the harsh conditions of coaxial interference, dramatically improves the stability of the system with a slight sacrifice in performance [15,16]. OA-ICOS overcomes these limitations by using an off-axis integrating cavity as the core component, resulting in improved detection strength and enabling global measurements [17,18,19].

It merits mentioning that owing to the design and operational requirements of OA-ICOS, the system is still susceptible to noise signals from electronics, optical interference, environment, and other sources. Traditional locked-in amplifiers incorporate wavelength modulation techniques to significantly improve the signal-to-noise ratio of the system [20,21,22]. However, these amplifiers require complex filtering algorithms, consume significant computational resources, and are not easily adaptable to versatile environments. On the other hand, machine learning, once it acquires the calibration data and is trained, only needs to perform matrix multiplication calculations to quickly generate detection results. Machine learning has been used as a concentration inversion algorithm in off-axis integrating cavities to optimize second harmonic filtering for CO_2_ detection; it improves the convergence accuracy of the algorithm [22]. But the researchers used machine learning only after the second harmonic had been extracted, causing some information to be lost in the process. In this study, machine learning has been utilized in an off-axis integrating cavity and used in the entire process from the detection of the signal to concentration inversion. The Extreme Learning Machine (ELM) has been trained using the original detector data, enabling it to account for even the smallest variations in the laser. This approach effectively enhances the extraction and utilization of information from the detector, improving instrument performance, including detection accuracy and limits.

## 2. Principles and Methods

### 2.1. Absorption Principles

#### 2.1.1. Laser Propagation Path

In OA-ICOS technology, a crucial component is the coaxial optical system, which consists of two highly reflective spherical mirrors known as an integrating cavity. When a laser beam interacts with a spherical mirror, it either converges or diverges, altering the beam’s direction. Under specific conditions, where the curvature radii of the two mirrors *R*_1_ and *R*_2_ and the cavity length *L* meet certain criteria, a paraxial beam can remain collimated after multiple reflections within the cavity and will not dissipate energy outside the cavity. The specific condition that the curvature radii *R*_1_, *R*_2_, and the cavity length *L* must satisfy to achieve this is given by the following expression:

(1) $$0<\left(1-\frac{L}{R_{1}}\right)\left(1-\frac{L}{R_{2}}\right)<1$$

This formula is also known as the resonant cavity stability condition, and a resonant cavity that satisfies this formula is referred to as a stable cavity.

When a laser beam penetrates the integrating cavity off-axis, it is reflected inside the cavity each time it hits the spherical mirror near the detector. Since the reflecting mirror is not infinitely large, the beam may exit the integrating cavity after a finite number of reflections, or it may reflect infinitely within the cavity, creating complex patterns of light spots on the reflecting mirror. This outcome is dependent on the incident point position of the laser beam, as well as the incident angles of the laser beam. When the curvature radius of the reflecting mirror and the cavity length satisfy the relationship described by Formula (1), the laser beam will create an elliptical light spot on the mirror’s surface based on the above parameters.

#### 2.1.2. Beer–Lambert Law

The intensity of a light beam will attenuate after it propagates through a medium, and this attenuation can be quantified using the expression given in Equation (2). This is known as the Beer–Lambert principle, which can be written as follows:

(2) $$I_{t}\left(\nu \right)=I_{IN\;}\left(\nu \right)e^{-\alpha \left(\nu \right)L}$$

where *I_t_* is the transmitted light intensity, *I_IN_* is the incident light intensity, *ν* is the wavelength of light in units of cm^−1^, *L* is the absorption path length in meters, and *α*(*ν*) is the absorption coefficient of the medium.

For a certain well-distributed gas, its absorption coefficient can be expressed using the following equation:

(3) $$\alpha \left(\nu \right)=\sigma \left(\nu \right)*N=NS\chi \left(\nu \right)$$

where *N* is the particle number concentration, *S* is the intensity of the absorption spectral line, and *χ*(*ν*) is the normalized line shape of the absorption spectral line. To determine the absorption cross-section of the gas at a specific wavelength in an optical system with a constant absorption path length, the outgoing light intensity can be measured and compared with the incident light intensity. This allows for calculating the concentration of the gas being measured.

#### 2.1.3. Principle of Integrating Cavity Output Spectrum

In several cases, the absorption cross-section of the gas being measured is too small, and concomitantly, the concentration is too low to induce a significant change in light intensity. In order to address this, the equipment needs to increase the optical path length, denoted as *L*. Researchers have proposed an integrating cavity output spectroscopy system to attain this goal without significantly increasing the complexity and volume of the optical system [15].

When a laser beam is incident into a resonant cavity that meets the conditions of Equation (1), it will undergo multiple reflections. For a reflecting mirror with a reflectivity less than 1 and using a dielectric film, a part of the energy not reflected will be transmitted outside the integrating cavity. Each time the laser energy is reflected, the under-investigated gas inside the cavity will absorb it.

The intensity of the light emitted for the first time is expressed as follows:

(4) $$I_{OUT1}=I_{IN}\left(1-R_{1}\right)\left(1-a_{absorb}\right)\left(1-R_{2}\right)$$

Thereafter, each time the laser beam reflects back and forth within the cavity, the energy emitted is ${\left(1-a_{absorb}\right)}^{2}R_{1}R_{2}$ times the previous one. The energy transmitted from the detector end lens for the *n*-th time is given by the following:

(5) $$I_{OUTn}=I_{IN}\left(1-R_{1}\right){\left(1-a_{absorb}\right)}^{2n+1}R_{1}^{n\;}\;R_{2}^{n}\left(1-R_{2}\right)$$

The total energy output after the laser beam passes through the integrating cavity is given by the following:

(6) $$I_{OUT}=I_{OUT1}+I_{OUT2}+\cdot \cdot \cdot \cdot \cdot \cdot +I_{OUTn}=I_{IN}\frac{\;\left(1-R_{1}\right)\left(1-R_{2}\right)\left(1-a_{absorb}\right)}{1-R_{1}R_{2}{\left(1-a_{absorb}\right)}^{2}}$$

For a cavity with no absorption, $a_{absorb}=0$, the total energy output after the laser beam passes through the integrating cavity is as follows:

(7) $$I_{0}=I_{IN}\frac{\left(1-R_{1}\;\right)\left(1-R_{2}\right)}{1-R_{1}R_{2}}$$

Under normal circumstances, the energy loss of the laser beam passing through the integrating cavity once is very small, and the absorption coefficient $a_{absorb}$ approaches zero. To achieve an increasing absorption path length, the reflectivity of the reflecting mirror is usually kept very high with the value of *R* approaching 1. Hence, the absorption coefficient can be approximately written as follows:

(8) $$\alpha \approx \frac{1}{d}\left(\frac{I_{0}}{I_{OUT}}-1\right)\left(1-\sqrt{R_{1}R_{2}}\right)$$

In comparison with the optical length of a single pass-through length d, the effective optical path can be expressed as follows:

(9) $$L_{eff}=\frac{1-\sqrt{R_{1}R_{2}}}{d}$$

#### 2.1.4. Selection of Absorption Lines

Carbon dioxide has three fundamental absorption bands located in the mid-infrared at $\nu_{1}=1337\;cm^{-1},\nu_{2}=667\;cm^{-1}$, and$\;\nu_{3}=2349\;cm^{-1}$, respectively. Although the absorption lines of the fundamental bands are relatively strong, it becomes challenging to manufacture lasers, optical fibers, and detectors corresponding to these wavelengths. Consequently, the overtone and combination bands are often used to detect methane concentration in the near-infrared region. For instance, distributed feedback (DFB) laser technology can easily produce lasers in the 1.57 µm or 2 µm band, and indium gallium arsenide detectors also possess a strong response in these bands. These two key devices can easily cover the $2\nu_{1}+\nu_{3}$ and $2\nu_{1}+{2\nu }_{2}+\nu_{3}$ combination bands of CO_2_. On the contrary, although the fundamental band $\nu_{3}$ provides absorption approximately 2744 times stronger than band $2\nu_{1}+\nu_{3}$ and about 203,448 times stronger than band $2\nu_{1}+{2\nu }_{2}+\nu_{3}$, the detection of mid-infrared gas spectra often necessitates expensive quantum cascade lasers (QCLs), interband cascade lasers (ICLs), and mercury cadmium telluride detectors [22,23].

**Figure 1:**
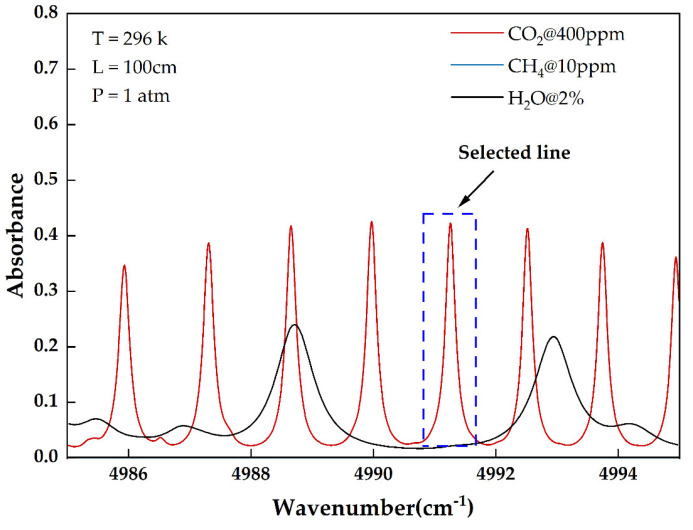
Absorbance of CO_2_ and common potentially disruptive components in 4987 cm^−1^ to 4995 cm^−1^ (from 2002.0 nm to 2005.2 nm, equivalently).

As shown in Figure 1, the absorption spectrum of common components in the air near 2004 nm at the $2\nu_{1}+\nu_{3}$ band is sourced from the HITRAN database. The absorption of the other main ingredients in the air in this band is much less than the absorption intensity of CO_2_ at this wavelength. The absorption at the corresponding concentration is less than 5% of the CO_2_ absorption. Any impact on the detection result can be corrected using technical means [24]. Therefore, 2004 nm has been selected as the absorption peak of the CO_2_ detection system.

### 2.2. The Principle of the Extreme Learning Machine

The Extreme Learning Machine (ELM) is a single-hidden layer feedforward neural network machine learning algorithm. The entire learning network is divided into three layers: the input layer, the output layer, and the hidden layer.

**Figure 2:**
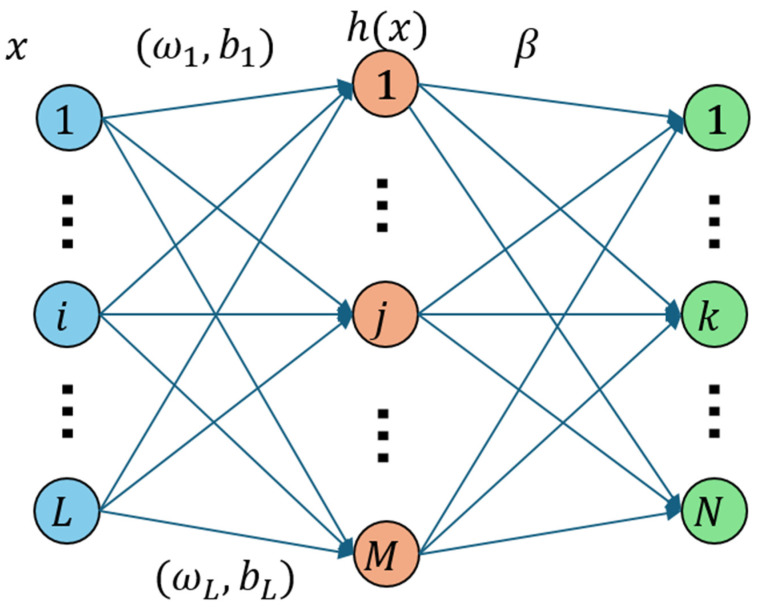
Schematic diagram of the Extreme Learning Machine model.

As exhibited in Figure 2, the model is fully connected from the input layer to the hidden layer and also fully connected from the hidden layer to the output layer. Assuming that the output of each neuron node in the hidden layer is $h_{1}\left(X\right),h_{2}\left(X\right)\ldots h_{M}\left(X\right)$ and denoting the output of the hidden layer as $H(X)=[h_{1}\left(X\right),h_{2}\left(X\right),\cdot \cdot \cdot ,h_{M}\left(X\right)]$, where *X* is a vector of a certain input layer. The model defines the output of the hidden layer as the result of the input layer multiplied by the corresponding weight *w_j_*, plus the corresponding bias *b_j_*, and then passed through a nonlinear function. This nonlinear function is also known as the activation function, which typically includes the Sigmoid function, Gaussian function, tanh function, and others [25,26].

(10) $$h_{j}\left(X\right)=g\left(w_{j}X+b_{j}\right),\;\;\;\;\;w_{j}\in R^{D},\;b_{j}\in R$$

In the end, the hidden layer enters the output layer after calculation:

(11) $$F\left(X\right)=H\left(X\right)\beta ,\;\;\;\;\;\beta \in R^{M\times N}$$

where *β* is the computational weight from the output layer to the hidden layer. In the above model, there are three unknown matrices: *w*, *b*, and *β*, which can be determined by entering the data of the training set. In the ELM model, *w* and *b* are randomly generated that are independent of the training data. Consequently, it is only necessary to find the weight matrix *β* that interconnects the hidden layer with the output layer using the following equation:

(12) $$\min\left|\left|H\beta -T\right|\right|$$

Through matrix theory, it is known that when $\beta =H^{+}\;T$, the above formula has an optimal solution, where $H^{+}$ is the Moore–Penrose pseudoinverse of matrix *H*. Upon successful execution of the training, the ELM model has all the parameters required for the calculation. By entering data in the same format as the training set, the output results can be obtained. This model can be applied to simultaneously perform the classification and regression tasks.

### 2.3. Sensor Configuration

**Figure 3:**
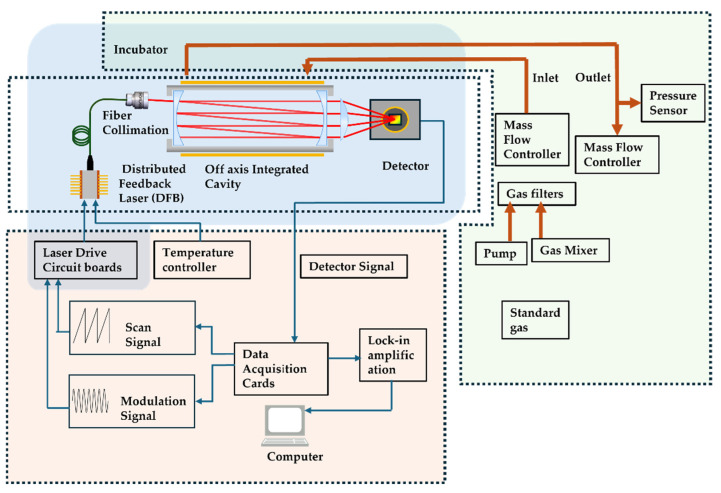
Schematic of carbon dioxide (CO_2_) detection system.

The schematic in Figure 3 depicts the structural layout of the carbon dioxide gas detector, which comprises optical, electronic, and environmental control components. The design of the gas absorption cell assists in determining the layout of the optical part. The orange section in Figure 3 represents the electronics part of the instrument. The electronics section is primarily responsible for controlling the laser, collecting detection signals, and calculating the gas concentration values. Under the control of the computer, the data acquisition card (USB-6211, National Instruments, Austin, TX, USA) outputs a sawtooth wave signal at a frequency of 10 Hz and superimposes a 2 kHz sine wave modulation signal. The laser driver board, to scan from 35 mA to 65 mA, controls the laser current. The thermistor and Thermo-Electric Cooler (TEC) inside the DFB laser(EP1573-0-DM-B01-FA, Eblana Photonics, Dublin, Ireland) are managed by the temperature controller, maintaining the internal temperature of the laser’s measurement point at 25 °C with an accuracy of 0.002 °C. This ensures circumventing any changes in the wavelength due to the thermal effect of the laser diode during operation. The laser signal received by the detector is converted into an analog voltage signal, which is then converted into a digital signal by the DAQ and input into the computer. The computer program further processes the ELM algorithm to obtain the concentration value of the sample gas. In this system, the modulation signal generation, detector signal acquisition, lock-in amplifier, cascade integrator comb (CIC) filter, and ELM are automated by a program based on the Matlab(R2016b) platform, which allows continuous, automatic, and full process operation.

The green section represents the environmental control component of the instrument, which regulates the temperature and pressure of the gasses inside the system. It is important to place key components into a constant temperature box to ensure the stable operation of the optical element. The components that should be placed in the constant temperature box are denoted by the blue frame and include a laser, gas absorption tank, collimator, and detector. The constant temperature box consists of a high-power TEC and a temperature sensor. The air temperature in the box is controlled to remain stable at 20 ± 0.02 °C through fan heat exchange. To facilitate the smooth entry of the sample gas into the absorption cell and maintain constant pressure, a mass flow controller (IKFD-LC-1000SCCM, AInuo Instrument, Qingdao, China) is connected before the gas enters the cavity. The mass flow controller can produce gasses of different concentrations by mixing carbon dioxide and nitrogen of standard concentration and delivers the gas into the cavity at a constant flow rate. If there is a need to measure the concentration of the actual sample gas, the valve of the mass flow controller can be closed, and the gas pump can be used for gas supply. Once the gas fills the absorption tank, it is released into the atmosphere through the second mass flow controller at the outlet. An absolute pressure sensor (CPT6100, MENSOR LP, San Marcos, TX, USA) is connected in the pipeline to monitor the real-time pressure change, and the opening of the second mass flow controller is adjusted accordingly to maintain constant pressure in the cavity.

The concentration of carbon dioxide in the atmospheric environment is about 400 ppm. Hence, to meet the system’s detection range and resolution requirements, two mirrors with a reflectivity of 99.90% @2004 nm (diameter 25 mm, curvature radius R1000, JGS3) are chosen to be installed at both ends of the gas absorption cell. The cell has a diameter of 3 cm, a length of 20 cm, and a volume of 141 mL. When paired with a flow pump of 1 L/min, this set up can achieve a response speed of 15 s. According to Formula (9), the equivalent optical path of the off-axis integrated cavity is 2.5 km.

Based on the selected absorption lines, additional key components have been designed for CO_2_ detection device. The core optical component of this device is the off-axis integrated cavity which is exhibited in Figure 3. The DFB laser emits light, with the center wavelength ranging from 2001.95 nm to 2005.33 nm, adjustable by modifying the current and temperature. The emitted beam passes through the collimator (F028APC-2000, Thorlabs Inc., Newton, NJ, USA) and then enters the off-axis integrated cavity. After multiple reflections and transmissions, the beam is focused by a lens and received by the PDA10DT detector (Thorlabs Inc., Newton, NJ, USA) on an image plane. This detector utilizes a transimpedance amplifier and includes an indium gallium arsenide photodiode with a 1 mm diameter image plane. When the maximum gain is 70 dB, the amplification factor can reach up to 4.75 × 106 V/A. The detector’s wavelength range is 900–2540 nm, and its response at 2004 nm is approximately 1.2 A/W. When driven by a sinusoidal modulation signal of 0, the detector picks up a direct absorption signal of CO_2_. By mixing CO_2_ with pure nitrogen at various concentrations, the integral of the absorbing area can be calculated to determine the equivalent optical range of the off-axis integrating cavity, which comes out to be 173 m. The designed reflector has a reflectivity of 99.90%, which gives a theoretical equivalent optical range of 200 m. However, due to challenges in perfectly matching the mode of the resonating cavity with the output laser beam and the presence of unavoidable interference noise, the actual optical range will be affected [21].

External vibrations, laser energy attenuation, fluctuations in the detector response, and other factors can have a significant impact on the results obtained through the direct absorption method. To enhance the signal-to-noise ratio of the signal and effectively detect small changes in gas concentration, a second harmonic, which involves wavelength modulation technique, is employed to the off-axis integrating cavity. This technique adds a high-frequency sinusoidal signal to the scanning signal. As long as the harmonic signal is not overwhelmed by noise, the gas absorption spectrum signal it contains can be distinguished.

## 3. Results

### 3.1. Gas Preparation

To evaluate the performance of the instrument, different concentrations of gasses were prepared. A bottle of high-purity nitrogen and a bottle of CO_2_ standard gas with an uncertainty of less than 0.5% at 1000 ppm were used. These gasses were passed through a mass flow meter to create various concentrations of CO_2_ at a constant flow rate of 500 mL/min. Eight concentrations of CO_2_ were prepared: 300 ppm, 350 ppm, 375 ppm, 400 ppm, 425 ppm, 450 ppm, 475 ppm, 500 ppm. The experiment began after flushing the gas absorption cell with the standard concentration gas. Care was taken to ensure collection of no less than 2000 points for each concentration at a sampling frequency of 10 Hz per second.

### 3.2. SNR Estimation

#### 3.2.1. Traditional Locked-In Amplifier

The conventional demodulation process relies on a quadrature locked-in amplifier that effectively extracts the signal at the target frequency. Studies have demonstrated that the amplitude of the second harmonic of the detection signal is directly proportional to the concentration of the gas being measured and is not affected by the absolute energy of the laser and detector. To process the detection signal, it passes through a locked-in amplifier with a two-fold sinusoidal modulation signal and then through a low-pass filter. Thereafter, the relative value of the gas concentration can be obtained. By using regression analysis in combination with the known gas concentration, a complete model of gas concentration and detection signal can be established. However, fluctuations caused by circuit noise and environmental factors have been minimized. Still, uncertain variations in the laser drive parameters and environmental parameters can affect the measurement results, causing changes in the half-height full width, peak value, center position, and symmetry of the second harmonic.

#### 3.2.2. Optimized CIC Filtering Scheme

After the locked-in amplifier, a low-pass filter is required to demodulate the original signal entirely. In digital systems, a finite impulse response (FIR) filter is commonly used for such processing. Experiments have indicated that the performance of the instrument is significantly impacted by the low-pass filter ability to eliminate high-frequency noise. On the other hand, the FIR filter’s ability to reject high-frequency signals is limited by the number of filter orders and the resources of the digital processor. Proper designing of FIR allows the cascade integrator comb (CIC) filter to achieve higher results than conventional FIR filters while utilizing fewer resources. While CIC filters offer significant advantages over conventional FIR filters in terms of noise suppression and reduced digital processor utilization, they introduce a greater signal delay, which can be mitigated by expanding the scanning range of the laser.

#### 3.2.3. Extreme Learning Machine

In this detection system, the signal-to-noise ratio is the ratio of the effective signal amplitude to the background noise. Both of the methods mentioned earlier can be used to experimentally obtain this parameter for evaluating the performance. However, since the machine learning model has only input and output variables, where the intermediate variables are derived from the spontaneous training of the model and lack practical significance, the traditional method of calculating the signal-to-noise ratio does not apply to machine learning and is not sufficiently discussed in this study. The performance of the CO_2_ detection system incorporating the ELM algorithm will be further investigated in the upcoming tests.

#### 3.2.4. Improvement of SNR of the Detection System

**Figure 4:**
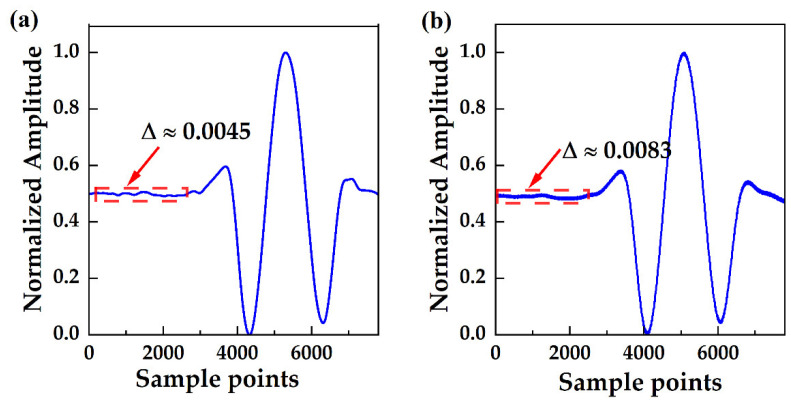
(**a**) Second harmonic of OA-ICOS at 400 ppm CO_2_ environment; (**b**) Second harmonic passing through the CIC filter of OA-ICOS at 400 ppm CO_2_ environment.

A number of factors affects the signal-to-noise ratio (SNR) of the detection system. In this study, the primary factors were the bandwidth noise of the laser, the interference noise of the gas absorption cell, the background noise of the detector, and the noise introduced by the amplification circuit. The SNR significantly affected the detection results. In OA-ICOS, the concentration of the gas to be measured was positively correlated with the peak value of the second harmonic. When the noise level was too high, the spectral information of the gas absorption was overwhelmed. SNR can be expressed as dividing the amplitude of the absorption spectrum by the amplitude of the non-absorption region. Even with the use of filtering, phase-locked amplification, and other technical means, the SNR remained a significant limiting factor in the detection results. Figure 4a shows the second harmonic of the normalized conventional phase-locked amplification. Figure 4b displays the second harmonic of the normalized combined CIC filter with phase-locked amplification. By comparing the standard deviation of the non-absorbing part of the spectra, signal-to-noise ratios of 120 and 220 can be obtained, respectively.

### 3.3. Signal Processing Performance of ELM

The ELM model needs to be trained with training set data before using machine learning. The detection signals of various concentrations of calibration gas outputs were fed into the input layer of machine learning. This process was carried out while keeping the environment unchanged. After training, all the parameters associated with the hidden junction layer were obtained to build up the machine learning model. Compared to the earlier methods, machine learning can fully utilize the information carried by the signal to produce more accurate results.

**Figure 5:**
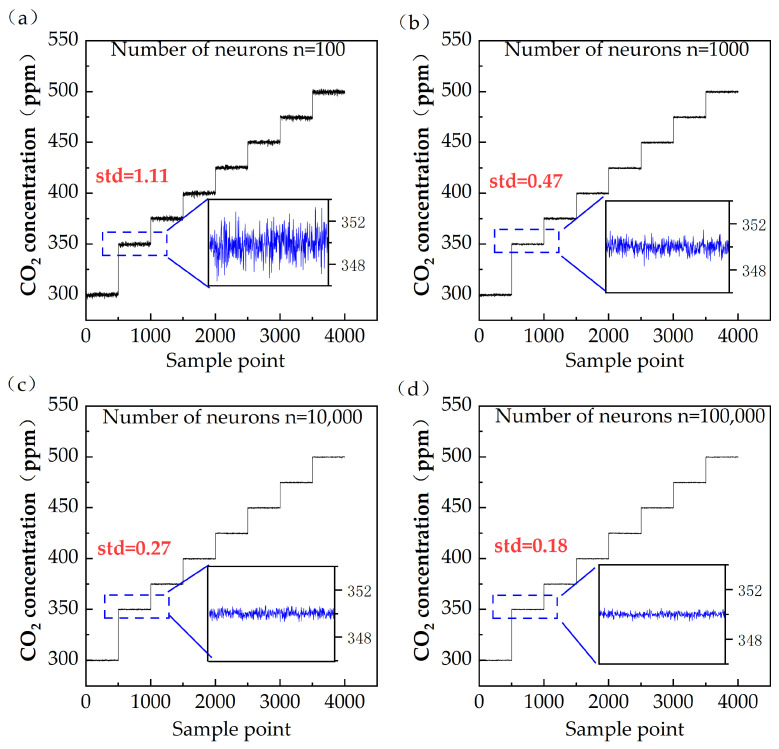
(**a**) Training results for ELM model with 100 neurons; (**b**) Training results for ELM model with 1000 neurons; (**c**) Training results for ELM model with 10,000 neurons; (**d**) Training results for ELM model with 100,000 neurons.

The system scanned the absorption spectrum of the gas at a frequency of 10 Hz, and the data acquired every second were combined in the time domain in order to eliminate high-frequency noise. A total of 600 s were sampled for each gas, of which the first 50 s data and the last 50 s data were eliminated to prevent the acquisition of data taken when the gas in the absorption cell had not stabilized during concentration switching. Repeating this process at eight concentrations resulted in a total of 500 data sets for each of the 8 concentrations. These data were used in the training set, where data were preprocessed with CIC filters before being passed to the machine learning input layer. Additionally, 500 data sets for each of the same 8 concentrations were used in the test set to evaluate the performance of the off-axis integral cavity carbon dioxide detection device. Two key parameters in the ELM model impact model building: the number of nodes in the input layer and the number of neural nodes in the hidden junction layer. The number of nodes in the input layer depend on the sampling frequency of the system and was determined in this model. It was found that neural nodes in the hidden junction layer significantly influenced the accuracy of the model. As evident from Figure 5, when the number of neural nodes in the hidden junction layer was taken as 100, 1000, 10,000, and 100,000, respectively, the fluctuation ranges of the measured concentrations of the trained models under 400 ppm CO_2_ test conditions were 6.72 ppm, 3.88 ppm, 1.09 ppm, and 0.81 ppm. The root-mean-square errors were 1.11 ppm, 0.47 ppm, 0.27 ppm, and 0.18 ppm, respectively. Similar trends were observed for the other concentrations.

With an increasing number of neural nodes, the model’s accuracy was significantly improved. However, having too many neural nodes used a lot of computational resources, leading to a significant increase in the model training and running times. Therefore, data preprocessing was necessary. Before feeding the data into the machine learning model, it underwent pre-filtering. The cascaded integrator comb (CIC) filter was used in this study. Choosing the appropriate extraction multiplicity not only reduces the amount of input data for the machine learning model but also pre-suppresses high-frequency noises in the signal. This leads to an enhancement in the input accuracy of the model and a reduction in the computational time consumed by the model.

**Figure 6:**
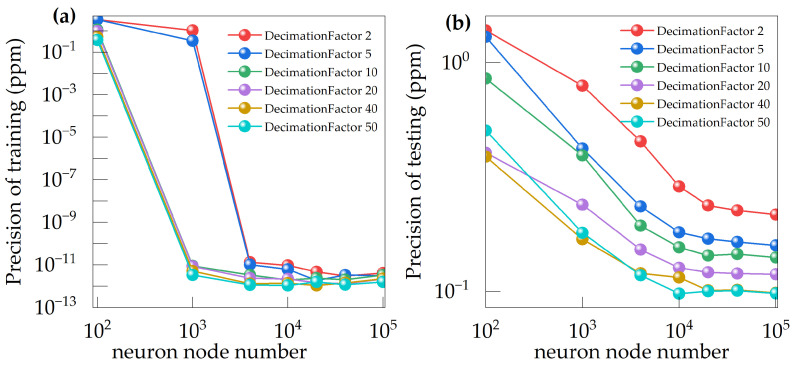
(**a**) Training set data precision of ELM model; (**b**) Testing set data precision of ELM model.

The graph in Figure 6 illustrates the training set data and testing set data accuracies of different decimation factors of CIC filter and limit learning machines with varying numbers of neurons. The findings indicate that increasing the number of neurons significantly improved training accuracy, and higher decimation factors also increased precision. However, it is important to note that model accuracy is limited and cannot be continually improved. Both the training and test data demonstrate the maximum limit in accuracy that could be attained. Beyond this point, increasing the extraction order of the CIC filter or the number of neurons in the machine learning model does not significantly improve the model accuracy. However, proper parameterization can help the model achieve optimal accuracy more efficiently without excessive consumption of the available resources.

**Figure 7:**
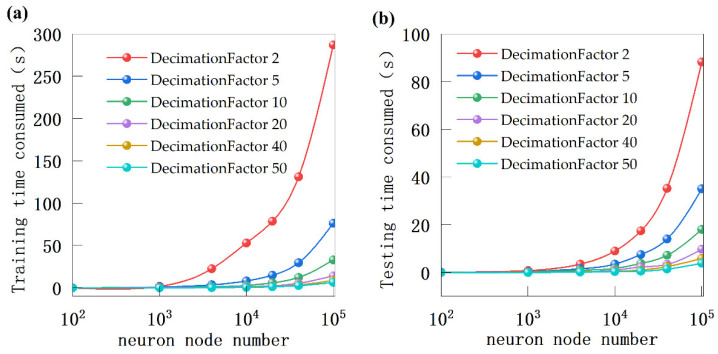
(**a**) Training time consumed of ELM model; (**b**) Testing time consumed of ELM model.

Nevertheless, increasing the number of neurons in a machine learning model also comes with additional complexities. Figure 7 illustrates the training and testing time of the model with varying numbers of neuron nodes and filter extraction multiples. Having too many neurons significantly increases the computation time for training and running the model due to computer resource constraints. It is crucial to consider both the accuracy and computational time consumption comprehensively, especially in scenarios with limited computational resources.

### 3.4. Fluctuation and Residual Analyses

**Figure 8:**
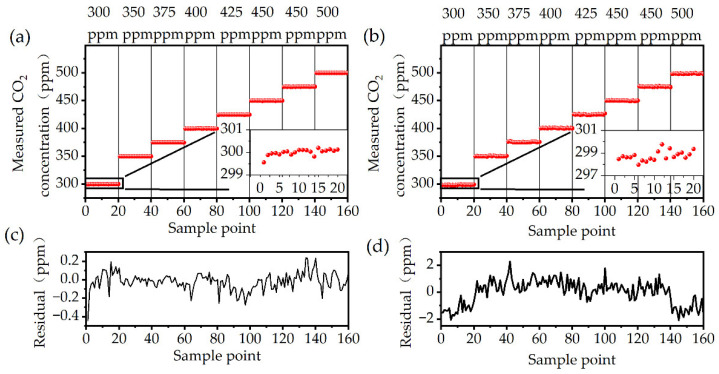
(**a**) Measurement results of cavity carbon dioxide detection system incorporating locked-in amplifier; (**b**) measurement results of dioxide detection system incorporating ELM; (**c**) residual of cavity carbon dioxide detection system incorporating locked-in amplifier; (**d**) residual of carbon dioxide detection system incorporating ELM.

As shown in Figure 8, in view of both the computational resources and associated performance, a 1000-neuron ELM machine learning model was employed to the CO_2_ detection system, which was passed through a CIC filter with an extraction factor of 20 prior to feeding the data to the input layer of the model. Experiments demonstrated that the CO_2_ detection system, incorporating a locked-in amplifier, had a maximum fluctuation of 4.53 ppm and a standard deviation of 0.97 ppm. In comparison, the CO_2_ detection system deploying a machine learning model had a maximum fluctuation of 0.65 ppm and a standard deviation of 0.16 ppm, resulting in a 6-times improvement in detection performance of the system.

### 3.5. Allan Deviation Analysis and Detection Limit

**Figure 9:**
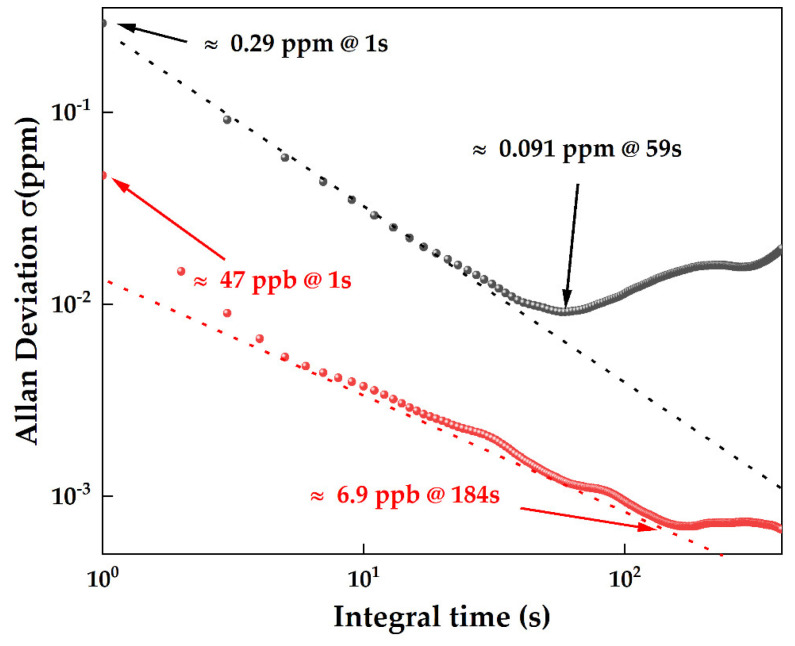
Comparison of Allan variance of cavity carbon dioxide detection system incorporating locked-in amplifier and dioxide detection system incorporating ELM.

Allan’s variance was originally used to estimate the frequency domain error in the field of inertial navigation. It represents the error level in different time scales and is commonly used in the field of gas detection to analyze instability in medium and long-term time scales. It is also used to estimate the detection accuracy of the gas concentration in different time scales. The analysis results in Figure 9 exhibit that the off-axis integrated cavity carbon dioxide detection system, which included a locked-in amplifier, had a lower detection limit of 0.29 ppm at an integration time of 1 s. The optimal integration time of 59 s was observed at which the lower detection limit was revealed to be 0.091 ppm. In contrast, the OA-ICOS dioxide detection system incorporating ELM machine learning model demonstrated a lower detection limit of 47 ppb at an integration time of 1 s. The optimal integration time of 184 s was observed at which the lower detection limit of 6.9 ppb was noted.

### 3.6. Field Test Results of Atmospheric CO_2_

**Figure 10:**
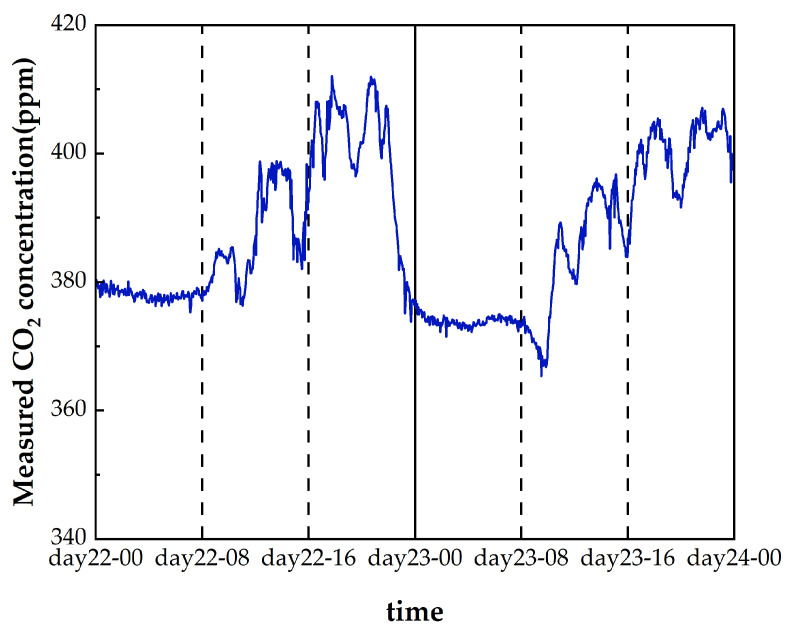
Measured urban atmospheric CO_2_ concentration.

As shown in Figure 10, a continuous urban atmospheric carbon dioxide concentration measurement experiment lasted for 48 h, from 23 February 2024 at 0:00 to 24 February 2024 at 23:59, and the equipment was set up at a height of about 45 m above the ground. The equipment was set up on the fifteenth floor of a building in the city at a height of about 45 m above the ground for continuous sampling, and the carbon dioxide concentration was stable and slowly decreasing in the early morning hours of 23 February and 24 February, whereas during the daytime hours of 23–24 February, the carbon dioxide concentration was at a relatively high level and fluctuated irregularly. There was also a clear diurnal cycle of different intensities and trends in human activities, such as transportation, industrial production, power generation, and other carbon emitting activities. We attribute the cause of this variation in measured atmospheric CO_2_ to these activities.

## 4. Discussion

Compared to the previously developed sensors using off-axis integral cavity output spectrum technology, this work compared the performance based on different signal processing methods. These methods include traditional locked-in amplifier, optimized locked-in amplifier, and Extreme-Learning-Machine-based processers. The ELM-based process exhibited significantly better performance in detection precision and detection limit but at the expense of a large number of training samples requirement.

This study combined the ELM algorithm and CIC filter to accurately detect the gas content present within the atmosphere. During the early stages of increasing the decimation factor of the CIC filter, a significant improvement in detection performance was observed. However, upon exceeding the decimation factor beyond 30, the rate of improvement slowed down. Additionally, increasing the number of neurons in the machine learning model from 100 to 100,000 also enhanced the detection performance. Furthermore, the experimental data related to detection precision and training consumptions provide a reference for making trade-offs in further design.

## 5. Conclusions

This paper described the design and implementation of an in situ trace gas sensor that utilized an ELM processor. The sensor in this work employed a machine learning model to effectively enhance its CO_2_ detection capability within an off-axis integrating cavity. The experimental results revealed that the use of an ELM processor can improve detection precision by six times compared to an Optimized CIC Filter locked-in amplifier. However, it is important to be cautious in seeking to enhance the performance of ELM processors due to the associated training costs. Through the use of a specifically designed gas cell, the detection limits for CO_2_ were determined to be 6.9 ppb at 184 s. The root-mean-square error (RMSE) of the test data set was observed to be 0.09 ppm. Future research will concentrate on finding a balance between optimizing training costs and performance, as well as integrating additional parameters into the machine learning model, such as ambient temperature, laser current, pressure of the gas absorption cell, and other variables.

## Data Availability

One part of the data presented in this study are openly available in Hitran at 10.1016/j.jqsrt.2021.107949, accessed on 5 November 2023, reference number [24].
